# Low-temperature plasma treatment induces DNA damage leading to necrotic cell death in primary prostate epithelial cells

**DOI:** 10.1038/bjc.2015.113

**Published:** 2015-04-02

**Authors:** A M Hirst, M S Simms, V M Mann, N J Maitland, D O'Connell, F M Frame

**Affiliations:** 1 York Plasma Institute, Department of Physics, University of York, Heslington, York YO10 5DD, UK; 2 Department of Urology, Castle Hill Hospital, Cottingham, East Yorkshire HU16, 5JQ, UK; 3 Hull York Medical School, University of Hull, Hull, East Yorkshire HU6 7RX, UK; 4 YCR Cancer Research Unit, Department of Biology, University of York, Heslington, York YO10 5DD, UK

**Keywords:** low-temperature plasma, necrosis, primary epithelial cells, prostate cancer, reactive species

## Abstract

**Background:**

In recent years, the rapidly advancing field of low-temperature atmospheric pressure plasmas has shown considerable promise for future translational biomedical applications, including cancer therapy, through the generation of reactive oxygen and nitrogen species.

**Method:**

The cytopathic effect of low-temperature plasma was first verified in two commonly used prostate cell lines: BPH-1 and PC-3 cells. The study was then extended to analyse the effects in paired normal and tumour (Gleason grade 7) prostate epithelial cells cultured directly from patient tissue. Hydrogen peroxide (H_2_O_2_) and staurosporine were used as controls throughout.

**Results:**

Low-temperature plasma (LTP) exposure resulted in high levels of DNA damage, a reduction in cell viability, and colony-forming ability. H_2_O_2_ formed in the culture medium was a likely facilitator of these effects. Necrosis and autophagy were recorded in primary cells, whereas cell lines exhibited apoptosis and necrosis.

**Conclusions:**

This study demonstrates that LTP treatment causes cytotoxic insult in primary prostate cells, leading to rapid necrotic cell death. It also highlights the need to study primary cultures in order to gain more realistic insight into patient response.

Despite continual improvement and refinement, long-term treatment for prostate cancer (PCa) is still recognised as inadequate (Jemal *et al*, 2011). In the case of early onset, organ-confined tumours, patients may be treated with a focal therapy (Kasivisvanathan *et al*, 2013; Donaldson *et al*, 2014). Radiotherapy or photodynamic therapy (PDT), which rely on the production of reactive oxygen species (ROS) for cytotoxic effects, are two treatments of choice for localised PCa. However, around a third of patients will experience recurrence of their disease following radiotherapy (Jones, 2011). This may be due to inherent radio-resistance of a small fraction of the tumour – the cancer stem-like cells (Frame *et al*, 2013). Furthermore, numerous side effects are often experienced following treatment (Chen *et al*, 2007; Lips *et al*, 2008), even with more recent technological developments, such as stereotactic body radiation therapy (Cyberknife) (Woo *et al*, 2014).

In recent years, low-temperature plasmas (LTPs) have shown considerable potential as active agents in biomedicine. They are formed by applying a high electric field across a gas, which accelerates electrons into nearby atoms and molecules, leading to a cascade effect of multiple ionisation, excitation and dissociation processes. This creates a complex and unique reactive environment consisting of positive and negative charges, strong localised electric fields, UV radiation, reactive species, and mainly background neutral molecules.

Operated at atmospheric pressure and around room temperature, LTPs produce high concentrations of reactive oxygen and nitrogen species (RONS), including but not limited to: atomic nitrogen (Wagenaars *et al*, 2012) and oxygen (Knake *et al*, 2008; Waskoenig *et al*, 2010; Niemi *et al*, 2013), hydroxyl (OH) (Ninomiya *et al*, 2013), singlet delta oxygen (SDO) (Sousa *et al*, 2011), superoxide (Kang *et al*, 2014), and nitric oxide (NO) (Ma *et al*, 2014). It is now widely believed that the principal mode of plasma–cell interaction is the delivery of RONS, a key mediator of oxidative damage and cell death in biological systems (Wiseman and Halliwell, 1996; Bandyopadhyay *et al*, 1999), generated in the plasma and transferred to target source (Graves, 2012, 2014).

In contrast, cell death by PDT relies on the generation of ROS, specifically SDO, which is highly cytotoxic (Sharman *et al*, 1999). Nonetheless, strong treatment resistance is encountered in hypoxic tumour regions (Krzykawska-Serda *et al*, 2014). The limitations of both radiotherapy and PDT, combined with the fact that LTPs concurrently produce both a multitude of RONS (Murakami *et al*, 2013) known to be toxic to cells and potentially strong localised electric fields, promotes the potential of LTP as a future cancer therapy, which we have recently reviewed (Hirst *et al*, 2014b).

Many studies now describe the effects of LTPs on various cancer cell lines in culture, with reported effects including reduced cell viability (Cheng *et al*, 2014; Plewa *et al*, 2014), growth arrest (Chang *et al*, 2014), and apoptotic cell death (Keidar *et al*, 2011; Gibson *et al*, 2014; Ishaq *et al*, 2014). We have reported induction of DNA damage by application of LTP treatment to primary prostate epithelial cells (Hirst *et al*, 2014a). Recent *in vivo* studies also revealed that LTP treatment of subcutaneous tumours (grown from cell lines) induced growth arrest and cell death, thus significantly reducing tumour volume in glioblastoma cells (Vandamme *et al*, 2012). Another study showed that short, daily exposure of tumours (squamous cell carcinoma) to LTP causes DNA damage leading to apoptosis (Kang *et al*, 2014). Internal treatment with LTP has also been explored using an endoscopic approach to application (pancreatic and colorectal cells), which demonstrated reduced tumour volume and also invasion capacity (Robert *et al*, 2013). However, the penetrative capability of LTP treatment through solid tissues leading to complete tumour eradication is yet to be established *in vivo*.

Here we first conducted a proof-of-principle study in order to validate the cytopathic effect of LTP treatment on two commonly used prostate cell lines derived from benign disease (BPH-1) and prostate cancer metastasis (PC-3). We then analysed in detail the effect of LTP treatment on a matched pair of primary samples. We cultured prostate epithelial cells from normal prostate and prostate cancer tissue (Gleason grade 7) retrieved from biopsies from a single patient, allowing for direct comparison of the effects of LTP on both normal and cancer cells. We present the first experimental evidence that LTP may be a suitable candidate for focal therapy treatment of patients with early onset prostate cancer through the induction of high levels of DNA damage, leading to a substantial reduction in colony-forming capacity, and ultimately necrotic cell death, in clinically relevant and close-to-patient samples.

## Materials and methods

### Culture of cell lines and primary prostate epithelial cells

Two prostate cells lines were used in this study: BPH-1 cells, derived from benign prostatic hyperplasia (BPH), and PC-3 cells, derived from PCa bone metastases. BPH-1 cells were cultured in RPMI 1640 medium supplemented with 5% foetal calf serum (FCS) and 1% L-glutamine. PC-3 cells were cultured in Ham's F12 medium, supplemented with 7% FCS and 1% L-glutamine. No antibiotic or antimycotics were added to the cell culture medium. Cells were incubated at 37 °C with 5% CO_2_.

Primary prostate epithelial cells were cultured from human tissue samples as described previously (Collins *et al*, 2005). Needle core biopsies (14 g) were taken immediately following surgical removal of the prostate. The site of each biopsy was determined by previous pathology, imaging, and palpation. Tissues were transported in RPMI-1640 with 5% FCS and 100 U ml^−1^ antibiotic/antimycotic solution at 4 °C and processed within 6 h. Needle core biopsies were verified as normal or Gleason grade 7 cancer by subsequent pathology, both cores originating from the same patient undergoing radical prostatectomy. Samples were obtained with full ethical permission and patient consent. Primary cells were cultured in stem cell media, based on keratinocyte serum-free media supplemented with L-glutamine, stem cell factor, granulocyte macrophage colony stimulating factor, cholera toxin, bovine pituitary extract, epidermal grown factor and leukaemia inhibitory factor (Collins *et al*, 2005). Significantly, these cells are cultured in media without FCS. Cells were grown on collagen-I-coated 10-cm dishes in the presence of irradiated STO feeder cells and incubated at 37 °C with 5% CO_2_. No antibiotic or antimycotics were added to the cell culture medium.

### LTP jet configuration and characterisation

**Figure 1:**
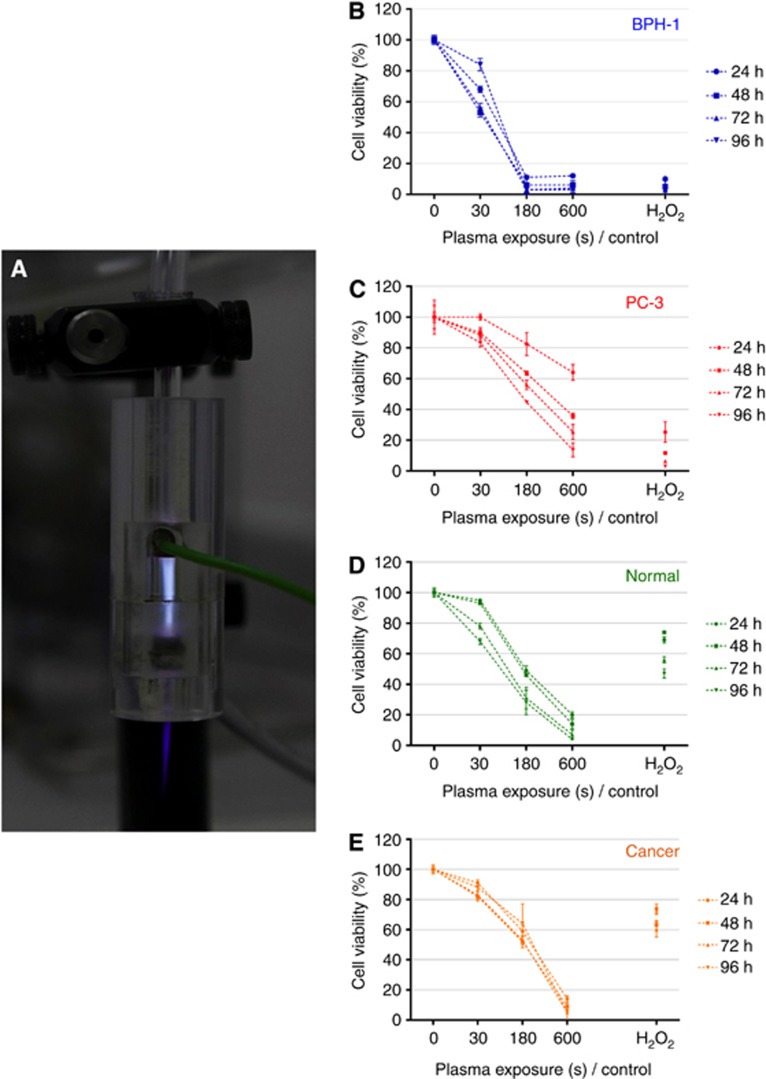
**LTP treatment leads to a reduction in cell viability.** (**A**) Cells were treated with a dielectric barrier discharge LTP jet, using 2 SLM helium flow rate with 0.3% O_2_ admixture, operated at 6 kV and 30 kHz. Reduction in cell viability was determined by alamarBlue assay (**B**, **C**) in BPH-1 and PC-3 cells and (**D**, **E**) in normal and cancer primary epithelial cells at 24, 48, 72 and 96 h postexposure.

The LTP jet consisted of a quartz glass tube of inner/outer diameter 4/6 mm, with two copper tape electrodes spaced 20 mm apart (Figure 1A). One electrode was powered (6 kV sinusoidal voltage at 30 kHz) and one grounded. Helium was used as a carrier gas at 2 standard litres per minute (SLM), fed with 0.3% molecular oxygen admixture. Cells were exposed to the LTP jet at a distance 15 mm from the end of the bottom electrode for a range of treatment times from 0 to 600 s in centrifuge tubes in a volume of 1.5 ml media. The distance between the end of the glass tube and the media surface was ∼2 mm. Hydrogen peroxide (H_2_O_2_, Fisher Scientific, Loughborough, UK) was used throughout as a positive cytoxicity control at a concentration of 1 mM. Using a thermocouple, treatment times of up to 600 s did not raise the surface temperature of culture media above 36.5 °C. The temperature and relative humidity of the laboratory were ∼20°C and ∼25% respectively.

Optical emission spectroscopy was performed using an Ocean Optics HR4000CG-UV-NIR spectrometer (Dunedin, FL, USA) (200–1100 nm range) and the Spectra suite analysis software (Dunedin, FL, USA). Integration time and scans to average were set at 6 and 50 s, respectively. A background dark spectrum was obtained and subtracted from subsequent spectra. The optical fibre was aligned directly with the core plasma region and fixed at ∼2 cm from the quartz tube.

### Cell viability and clonogenic recovery assays

Cell viability was determined by use of the alamarBlue assay (Invitrogen, Life Technologies Ltd, Paisley, UK). Cells were treated with LTP and then plated into black-walled 96-well plates in triplicate at a density of 5000 cells per well in 100 *μ*l of media. At 24, 48, 72, and 96 h after treatment, 10 μl of alamarBlue reagent (DAL1025, Invitrogen) was added to each well and incubated for 2 h at 37 °C. Fluorescence was recorded at excitation/emission values of 544/590 nm using a microplate reader (Polarstar Optima, BMG Labtech, Aylesbury, Bucks, UK), with cell viability recorded against normalised untreated samples.

Clonogenic recovery assays were used to measure cellular recovery posttreatment. Cells were treated in suspension and replated in six-well plates in triplicate at a density of 200 disaggregated cells per well. Cells were supplemented with 2 ml of growth media, which was changed every other day. In the case of primary epithelial cell cultures, STO feeder cells were also added. At 12 days after treatment, plates were stained with crystal violet (PBS, 1% crystal violet, 10% ethanol). Only colonies of >50 cells (equating to >5 population doublings) were counted (Francipane *et al*, 2008).

### DNA damage

LTP-induced DNA damage was quantified using the alkaline comet assay (adapted from Sturmey *et al*, 2009). Cells were treated with LTP in 1.5 ml centrifuge tubes at a density of 20 000 cells in 1.5 ml media suspension. Immediately after treatment, cells were resuspended in 30 *μ*l PBS and mixed with 225 *μ*l low melting point agarose. This was then pipetted onto microscope slides precoated with high melting point agarose and placed into lysis buffer (2.5 M NaCl, 10 mM Tris, 1 mM EDTA, 10% DMSO, 1% Triton X-100), overnight at 4 °C. The following day, cells were placed in alkaline buffer (0.3 M NaOH, 1 mM EDTA, pH 13) on ice for 40 min, before being electrophoresed at 23–25 V, 300 mA in alkaline buffer for a further 40 min on ice. Slides were then placed into neutralising buffer (0.4 M Tris, pH 7.5) for 2 × 10 min, before DNA was stained using SYBRgold (1 : 10 000 in TE buffer: 10 mM Tris, 1 mM EDTA, pH 7.5). Images were acquired by fluorescence microscopy (Nikon Eclipse TE300 microscope (Nikon, Surrey, UK), × 10 objective lens) using Volocity software (Volocity 6.3, PerkinElmer Inc., Waltham, MA, USA). Autocomet software (Tritek Corp., Sumerduck, VA, USA) was used to analyse cell images, with the median percentage of DNA-in-tail values used to record DNA damage in a minimum of 100 cells per treatment.

### Detection of ROS

Extracellular H_2_O_2_ formed in the culture media as a result of LTP treatment was detected and quantified using the ROS-Glo H_2_O_2_ assay (Cat. no. G8820, Promega, Southhampton, UK). Cells were treated with LTP, before being plated into black-walled 96-well plates at a density of 10 000 cells in 80 *μ*l of culture media, before following the manufacturer's protocol. Luminescence intensity was quantified using a microplate reader (Polarstar Optima, BMG Labtech) and normalised to untreated wells.

### Western blotting

Cells were collected at 2, 4, 8, and 24 h following LTP treatment, and protein lysates were extracted using Cytobuster Protein Extraction Reagent (71009, EMD Millipore, Darmstadt, Germany) with protease inhibitors (cOmplete, EDTA-free Protease Inhibitor Cocktail Tablets, Roche Applied Science, Burgess Hill, West Sussex, UK). Primary antibodies included: cleaved-PARP (Asp214, 1 : 666, Cell Signalling Technology Inc., Hitchin, UK; no. 9541S), anti-LC3B (Ab51520, Abcam, Cambridge, UK; 1 : 3000), and monoclonal anti-*β*-actin 1 : 5000 (A5316, Sigma-Aldrich, Gillingham, UK; mouse). Secondary antibodies used included: anti-rabbit IgG HRP-linked (1 : 5000, Cell Signalling Technology Inc. no. 7074S), and anti-mouse IgG peroxidase (1 : 5000, Sigma-Aldrich A5906). Staurosporine (1 *μ*M) was used as a positive control for apoptosis (Cell Signalling Technology Inc. no. 9953). Kaleidoscope protein ladder was used as a marker for all gels (161-0375, Bio-Rad). Staurosporine (1 *μ*M) was used as a positive control for apoptosis (Cell Signalling Technology Inc. no. 9953). The ratio of LC3BII/I band intensity was performed using ImageJ software (Mount Royal, QC, Canada), with all bands quantified against *β*-actin loading control lanes and normalised untreated controls.

### Caspase-Glo 3/7 assay

Cells were treated with LTP and plated into collagen-coated 96-well plates at a density of 20 000 cells per well in 100 *μ*l. A further 100 *μ*l of caspase-glo 3/7 detection reagent (Cat. no. G8090, Promega) was immediately added to each well. Cells were incubated at 37 °C, with luminescence intensity (Polarstar Optima, BMG Labtech) recorded at 24 h after treatment. Based on findings from other results, a reduced set of LTP exposures was used for this assay.

### CellTox necrosis assay

LTP-induced necrosis was quantified using the CellTox green cytoxicity assay (Cat. no. G8741, Promega). Cells were treated with LTP and plated into collagen-coated black-walled 96-well plates at a density of 10 000 cells in 50 *μ*l of media per well. In addition to H_2_O_2_ and staurosporine, 2 *μ*l of lysis solution (supplied with assay) was added to necrotic control wells. Fluorescence intensity was recorded using a plate reader (Polarstar Optima, BMG Labtech), at excitation/emission wavelength 485/520 nm, with readings at 2, 4, 8, 12, and 24 h after treatment. Fluorescence was normalised to untreated wells. Complementary fluorescence-brightfield merged microscopy images were also taken (Nikon Eclipse TE300 microscope, × 10 objective lens) at the same posttreatment time intervals.

### Statistical analysis

All experiments were performed in triplicate, and results are expressed as the mean with associated s.e., with the exception of comet assay data, which shows the median DNA damage value. Plots were constructed and statistical analyses performed using Prism 6 (GraphPad software, San Diego, CA, USA). Statistical significance was determined using unpaired Mann–Whitney test (DNA damage results only) or unpaired *t*-test with Welch's correction (assumes non-equal s.d.) and displayed on figure plots as *P<0.05, **P<0.01, ***P<0.001, and ****P<0.0001.

## Results

### Reduction in cell viability is observed following LTP treatment

The viability of cells was quantified at 24, 48, 72, and 96 h following LTP treatment (Figure 1). A reduction in viability in both BPH-1 (Figure 1B) and PC-3 (Figure 1C) cell lines was observed. BPH-1 viability was reduced to <20%, whereas viability of PC-3 cells was reduced to <40%. In addition, reduced cell viability was recorded in both normal and primary cells (Figure 1D and E), with 30-s LTP exposure leading to a small decrease in viability and 600-s exposure reducing cell viability to <20%. The positive H_2_O_2_ control was less toxic to both primary samples than the longer LTP exposures (180 and 600 s). The cell lines were more susceptible to 1 mM H_2_O_2_, (up to 90% reduction) than primary cells, which only had a ∼30% reduction with H_2_O_2_ alone. Furthermore, the duration posttreatment had little effect on viability in primary samples, with comparable results recorded at 24 and 96 h, particularly in the tumour cells (Figure 1E).

### DNA damage is sustained as a result of LTP exposure

**Figure 2:**
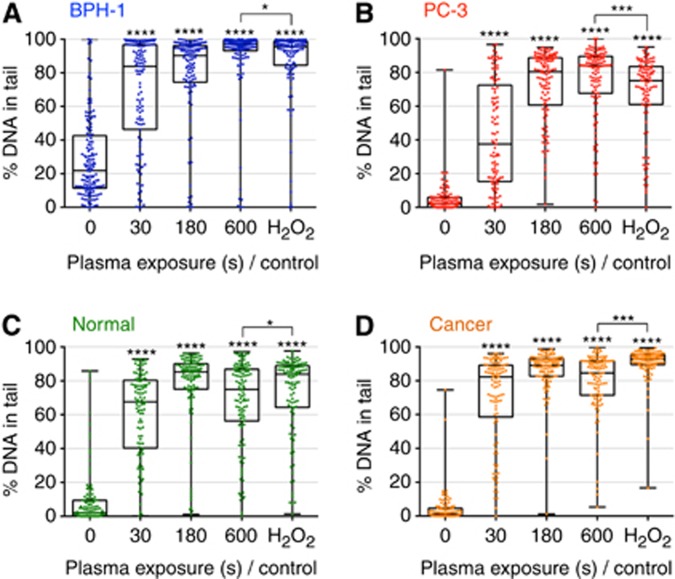
**LTP treatment induces high levels of DNA damage in both prostate cell lines and primary epithelial cells.** Cells were treated with LTP or H_2_O_2_ control (1 mM). DNA damage levels were measured using the alkaline comet assay and are represented as the percentage of DNA-in-tail in (**A**) BPH-1 and (**B**) PC-3 cells and in (**C**) normal and (**D**) cancer primary epithelial cells. Each dot represents a single cell, with a minimum of 100 cells counted for each exposure. Data are expressed as median±s.e. and are analysed by Mann–Whitney test. All significance was determined against untreated samples unless otherwise indicated.

LTP-induced DNA damage was assessed using the alkaline comet assay, with the percentage of DNA-in-tail recorded for analysis. Figure 2A and B show the percentage of DNA damage in both BPH-1 and PC-3 cells, respectively, for various exposure times. Each dot represents the DNA-in-tail percentage value from a single cell. Exposures as short as 30 s induced high levels of DNA damage, with a saturation of damage levels occurring from 180 s. This concurs with findings in normal and tumour primary cells (Figure 2C and D). The level of DNA damage from LTP exposure was found to be consistently comparable to the H_2_O_2_ treatment control, and the level of damage in the tumour-derived primary sample (Figure 2D) was marginally higher (but statistically significant, *P*<0.001) than that recorded for the normal sample (Figure 2C).

### Inhibition of colony-forming capacity as a result of LTP treatment

**Figure 3:**
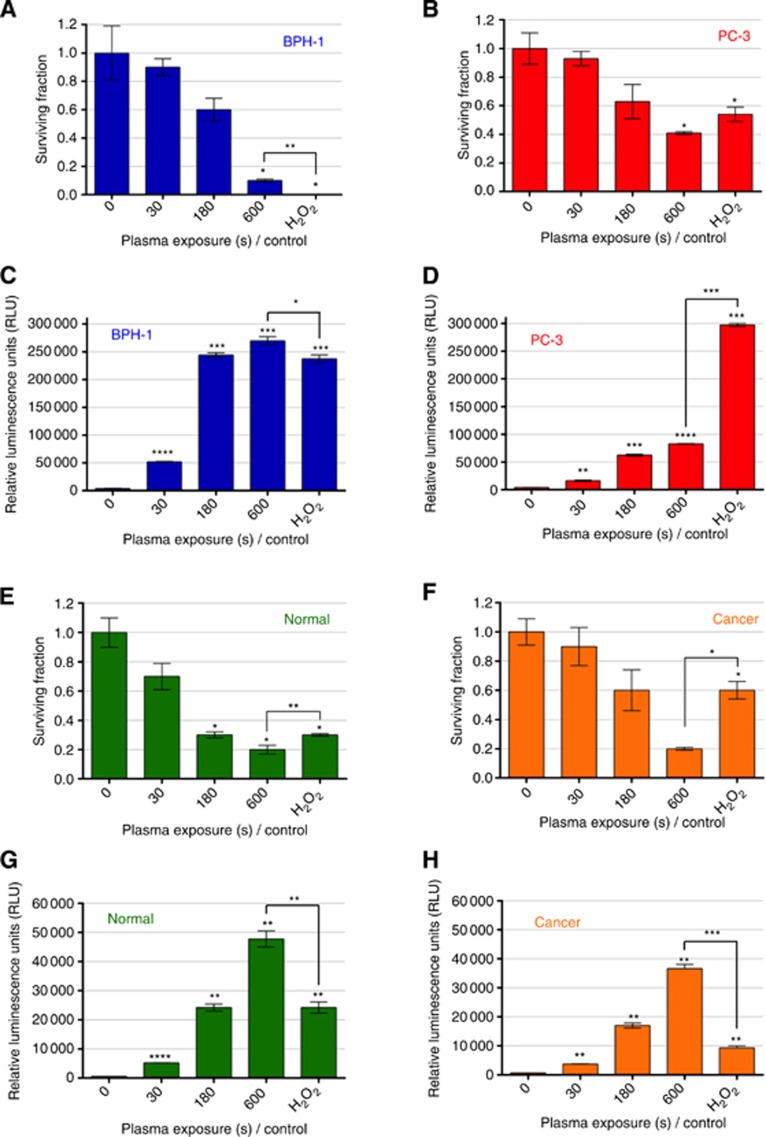
**LTP treatment leads to reduction in colony-forming efficiency, and high levels of H_2_O_2_ in cell culture media.** Cells were treated with LTP or H_2_O_2_ control (1 mM). Cell recovery was quantified using the colony-forming assays and is represented as surviving fraction posttreatment in (**A**) BPH-1 and (**B**) PC-3 cells and in (**E**) normal and (**F**) cancer primary epithelial cells. Immediately following treatment, ROS-Glo H_2_O_2_ luminescence assay (Promega) was performed to ascertain H_2_O_2_ levels in the culture media of (**C**) BPH-1 and (**D**) PC-3 cells and in (**G**) normal and (**H**) cancer primary epithelial cells (Note the different *y* axis scales). Data are expressed as mean±s.e., with statistical analysis conducted using unpaired *t*-test with Welch's correction. All significance was determined against untreated samples unless otherwise indicated.

Treatment with LTP showed a dose dependent inhibition of cell recovery in both BPH-1 and PC-3 cells, with the cancer cell line being more resistant than the benign cell line (Figure 3A and B). Findings in primary cells showed that treatment with 600-s LTP reduced the surviving fraction to ∼20% in both normal and tumour samples (Figure 3E and F). The tumour cells appeared significantly more resistant to the shorter 180-s LTP exposure and to the H_2_O_2_ control than the normal cells.

### Evaluation of H_2_O_2_ formation in cell culture media

Cells in suspension were treated with LTP for a variety of times before being analysed for the presence of extracellular H_2_O_2_ in the cell culture media, as an indication of LTP-induced ROS production. It is well known that H_2_O_2_ is extremely toxic to cells (Bandyopadhyay *et al*, 1999), even at micromolar concentrations (Gulden *et al*, 2010). Figure 3C and D show an increase in the relative concentrations of H_2_O_2_ generated in the culture media with increasing LTP exposure times for BPH-1 and PC-3 cells. H_2_O_2_ production in PC-3 cells was far lower following LTP compared with H_2_O_2_ control, yet 180 and 600-s LTP exposures in BPH-1 cells were comparable to H_2_O_2_ control. In primary samples, a treatment time of 180 s yielded luminescence values comparable to 1 mM H_2_O_2_ positive control in normal cells. An exposure time of 600 s corresponded to approximately two-fold of the concentration of a 1 mM H_2_O_2_ positive control in normal cells and three-fold in cancer cells (Figure 3G and H).

### LTP exposure induces different cell death pathways in cells lines and primary prostate epithelial cells

**Figure 4:**
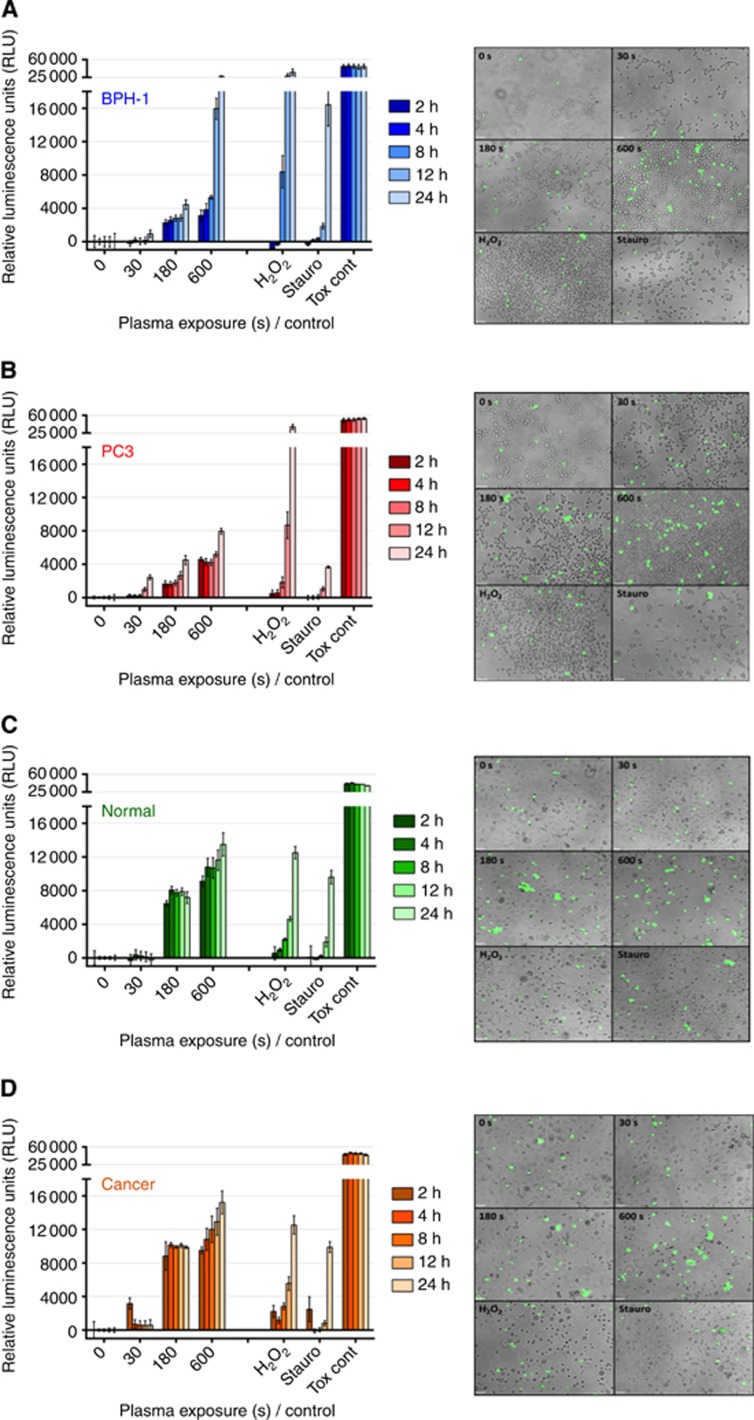
**LTP treatment leads to necrotic cell death in both prostate cell lines and primary epithelial cells.** Immediately following LTP treatment, the CellTox green cytoxicity assay (Promega) was performed to identify cells with comprimised membrane integrity characteristic of necrosis. In all, 1 mM H_2_O_2_, 1 μM staurosporine, and cell lysing agent (supplied with assay) were used as controls. Fluorescence intensity was quantified at 2, 4, 8, 12, and 24 h following treatment and normalised to untreated control wells in (**A**) BPH-1 and (**B**) PC-3 cell lines and in (**C**) normal and (**D**) cancer primary epithelial cells. Data are expressed as mean±s.e. Supporting fluorescence microscopy images ( × 10 magnification) taken at 4 h after treatment are also shown for each cell type.

Our results indicate that LTP exposure causes necrosis in both BPH-1 and PC-3 cell lines, as seen in Figure 4A and B. It is clear that PC-3 cells are more resistant to LTP-induced necrosis than BPH-1 cells. Significantly, necrotic cell death was also observed in both normal and cancer prostate primary cells. Figure 4C and D indicate that 180- and 600-s LTP exposures lead to high levels of necrosis compared with untreated control, with the findings common to both normal and cancer samples. This was verified by supportive fluorescence images taken at 4 h after treatment, which highlighted cells positive for necrosis (green) in 180- and 600-s LTP exposure wells, whereas staurosporine controls were largely negative at this time point.

Initially, the levels of necrosis at 2 h posttreatment were comparable for 180- and 600-s exposures. Cells treated with 600-s LTP showed a marked time-dependent increase in necrosis, whereas values for 180-s samples remained approximately constant. Interestingly, cytopathic effects with the pure H_2_O_2_ control did not present until around 12–24 h after treatment. Likewise, the staurosporine treatment induced necrosis only at 24 h, indicative of late-stage apoptosis.

**Figure 5:**
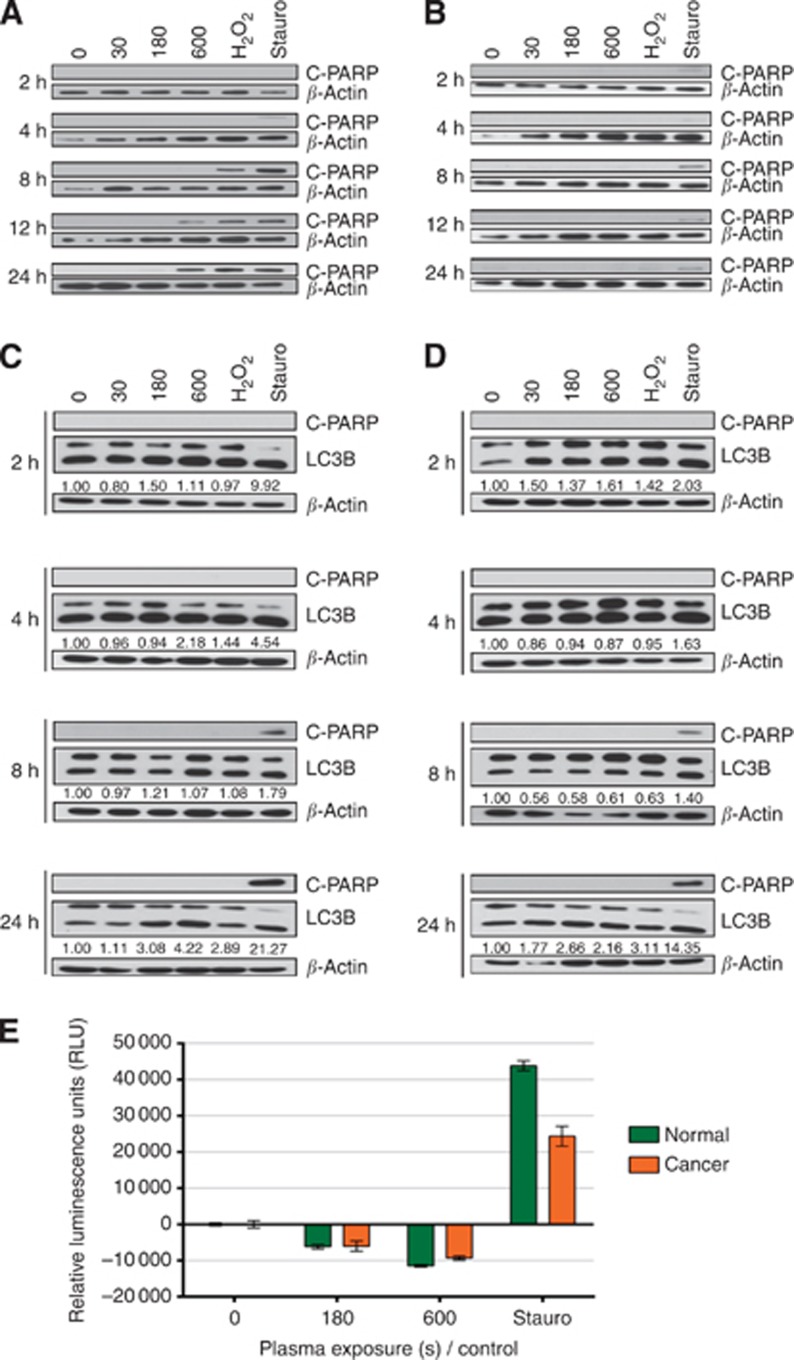
**Cell death mechanisms following LTP treatment vary between cell types.** Following treatment with LTP, 1 mM H_2_O_2_, or 1 μM staurosporine, protein lysates were harvested. (**A**) BPH-1 and (**B**) PC-3 cell line lysates were probed for apoptosis (C-PARP) by western blotting. (**C**) Normal and (**D**) cancer primary epithelial lysates were probed for apoptosis (C-PARP) and also autophagy (LC3B I/II). *β*-Actin was used as a loading control throughout. Band intensity quantification was performed using the ImageJ software. Further analysis of apoptotic activity was conducted in (**E**) primary epithelial cells using Caspase-glo 3/7 assay (Promega). Immediately following treatment, caspase-glo 3/7 detection reagent was added to all wells, and luminescence intensity was quantified at 24 h. Readings were normalised to untreated control and are expressed as mean±s.e.

In addition to necrosis, a proportion of BPH-1 cells also underwent apoptosis following LTP exposure as verified by western blotting for the presence of cleaved-PARP, whereas PC-3 cells did not (Figure 5A and B). Primary cells treated with LTP did not undergo apoptosis (Figure 5C and D). This was further confirmed by assessment of caspase 3 and 7 activity in primary samples (Caspase-glo 3/7 assay, Promega), where only staurosporine-treated positive control cells showed positive expression (Figure 5E). Indeed, LTP-treated primary cells showed apoptotic activity levels below those of untreated control, further verifying that cell death following LTP exposure occurs through necrosis and not apoptosis.

In addition to apoptosis and necrosis, another cellular response to stress is autophagy, which can serve as a protective mechanism, but also results in cell death. Quantitation of LC3 II/I band intensity revealed that, by 24 h posttreatment, a more than two- and a more than four-fold increase (over untreated controls) was present in the cancer and normal samples, respectively, indicating that an autophagic response occurred following LTP exposure (Figure 5C and D).

## Discussion

**Figure 6:**
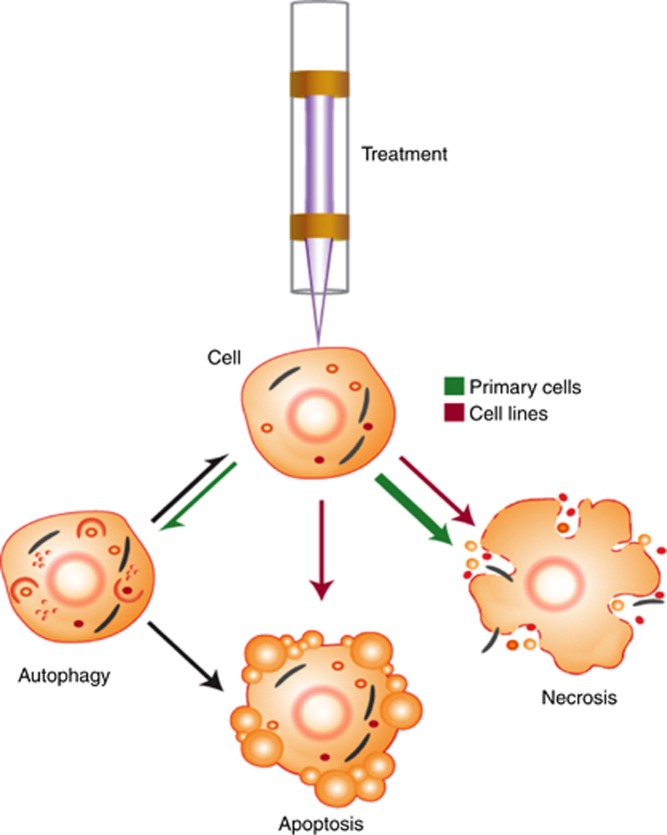
**Overview of cellular response mechanism following LTP treatment.** As a result of exposure to LTP, cells were observed to undergo (or a combination of) autophagy, apoptosis or necrosis. The relative proportions of, and differences between, cell lines (red arrows) and primary epithelial cells (green arrows) that exhibit these phenomena is emphasised. Adapted from Kepp *et al* (2011).

In this work, we have shown that treatment with LTP causes DNA damage, a reduction in both cell viability and recovery, and ultimately necrotic cell death in normal and cancer primary prostate epithelial cells. The results indicate that LTP-induced H_2_O_2_ in the culture media is a likely facilitator of these effects. We also observed that, unlike primary cells and the PC-3 cell line, BPH-1 cells also die through apoptotic mechanisms following plasma treatment (Figure 6). Our findings in primary cells highlight the potential of LTP as an alternative to, or for use in conjunction with, other existing treatments for organ-confined prostate cancer. Furthermore, the differential cell death response between cell lines and primary cells stresses the need to study clinically relevant models in order to gain insight into the potential patient response.

LTP exposure is known to cause cytotoxic effects in cells via the delivery of RONS to the liquid environment (Ahn *et al*, 2014; Ishaq *et al*, 2014; Ma *et al*, 2014). Our results indicate that 180-s LTP treatment of prostate primary cells leads to H_2_O_2_ concentrations approximately equal to that of a 1 mM H_2_O_2_ control. Additionally, LTP exposure of 600 s produced statistically significant H_2_O_2_ readings two- to three-fold to those of the control. Interestingly, following exposure to LTP, the levels of H_2_O_2_ recorded in the tumour cells were found to be lower generally than those from the normal cells, resulting in an enhanced colony recovery following treatment at 30- and 180-s LTP treatment times but not at the longest exposure of 600 s. This is in keeping with recent data, suggesting that cancer cells have the ability to quench the effects of ROS more effectively than normal cells (Diehn *et al*, 2009; Gorrini *et al*, 2013). Despite this, cell viability is still strongly reduced following LTP treatments of 180 and 600 s, indicating that any RONS produced initially in the culture medium remain strongly damaging to the primary cells at increased time periods postexposure. In contrast to data from the proof-of-principle study on prostate cell lines (Figure 1B and C), the primary samples appear far more resistant to the H_2_O_2_ control, yet the reduction in viability as a result of LTP exposure is comparable between the different samples (Figure 2C and D). This suggests that the enhanced effect of the plasma is likely to be due to a cumulative effect on the cells of a multitude of reactive species produced in the plasma (the presence of atomic oxygen in the plasma core was verified by optical emission spectroscopy, Supplementary Figure S1), and/or additional plasma components such as electric fields, charges, and UV radiation (Graves, 2012; Kang *et al*, 2014), rather than just solely due to H_2_O_2_. This may also make it unlikely for cancer cells to become resistant to treatment, as increased tolerance to a particular reactive species would not protect against the perceived multi-faceted action of LTP. Because of the added presence of reactive nitrogen species produced by some LTPs (Cheng *et al*, 2014; Gibson *et al*, 2014), this may also present an advantage over radiotherapy, which relies heavily on ROS alone (Palacios *et al*, 2013), and over PDT, which relies predominantly on the single reactive species SDO for its cytotoxic effect (Sharman *et al*, 1999).

A contribution of the cell culture media to the observed effects cannot be discounted. We measured a three-fold increase over control of H_2_O_2_ production after plasma treatment in primary cells. Yet, we see that, in the BPH-1 cell line, the LTP-treated H_2_O_2_ concentrations are broadly similar, and in the PC-3 cell line the H_2_O_2_ concentrations are much lower than the control (Figure 1E and F). It is known that different cell culture media can produce different amounts of H_2_O_2_ (Promega Technical Services, private communication). We therefore considered treating all cell types in a buffered saline solution and re-plating the cells in their optimal culture media. However, a counter-argument is that this would not have been physiological (with respect to treating a patient) and that any cytopathic effect would be likely to be predominantly due to short-lived reactive species, and the prolonged effects of long-lived species would be lost. Significantly, both normal and cancer primary cells used in this study were cultured and treated in identical media without serum, and so media was not a variable factor and the results from these cells can be directly compared.

Differences in H_2_O_2_ levels were recorded in treated media containing cells and treated media only. All plasma-treated samples showed a reduction in H_2_O_2_ production in the presence of cells (*vs* treated media), suggesting that the cells consume, or quench, H_2_O_2_ in the media (Supplementary Figure S2A). This was by far the most pronounced in primary cells, where the H_2_O_2_ level following 180-s LTP exposure was reduced by 78% in the presence of cells. There was far less of a reduction in BPH-1 cells (17%) and PC-3 cells (41%). It was also found that, by 2 h following treatment, the levels of H_2_O_2_ (induced by either 600-s plasma treatment or 1 mM H_2_O_2_) were strongly reduced in both normal and tumour primary cells. This effect was more pronounced in the tumour cells and demonstrates the strong ROS-quenching capacity of the primary cells (Supplementary Figure S2B and C). The level of H_2_O_2_ formed by the positive control was further reduced to that of the untreated cells by 8 h; however, there were still elevated levels of H_2_O_2_ induced by plasma treatment detected at this time point.

We have found that high levels of DNA damage, which is uniform across all cell types, is inflicted after an LTP exposure of only 30 s. In addition, a reduction in colony-forming ability following LTP treatment was observed, as cells treated with 600-s LTP recovered significantly less than those treated with the H_2_O_2_ control. This is despite the DNA damage values between 600 s and H_2_O_2_ control differing by only a few percent across all samples, in support of the hypothesis that the cytocidal effect of the plasma on cells is not solely due to H_2_O_2_ production. Therefore, *in vitro*, retaining the cells in treated media is necessary to realise a strong anti-proliferative effect (which we investigated and found to be the case; data not shown), as would be seen in tissues. Other LTP-based studies report a selective plasma effect (Wang *et al*, 2013; Guerrero-Preston *et al*, 2014), that is, that the plasma preferentially induces cell death in cancer cells. However, normal and tumour cell lines studied often originate from different sites or hosts or are cultured in different media. We observe similar responses in both primary prostate tumour and normal cells from the same patient, highlighting the necessity for supporting live imaging, for example, MRI, for precise targeted tumour ablation in patients (Sullivan and Crawford, 2009).

Finally, for any progression towards a patient therapy, further elucidation of the mechanism of LTP-induced cell death is required. Following a fatal stimulus, cell death can occur broadly in one of the two ways; apoptosis – a regulated chain of events involving cell shrinkage, blebbing, and ending with the formation of apoptotic bodies that retain membrane integrity (Cohen, 1997), or necrosis – an uncontrolled swelling that leads to membrane rupture and spillage of the cell contents into the surrounding environment, provoking an inflammatory response (Casiano *et al*, 1998). It is clear from our results that primary cells rapidly undergo necrosis, in the almost complete absence of apoptosis. A major advantage of this is that necrotic cell death has the potential to promote immune-activation against tumour cells (Melcher *et al*, 1999). In contrast, apoptotic cell death has been observed to promote an immune-suppressive environment (Voll *et al*, 1997), allowing tumour cells to evade detection by the immune system (Gregory and Pound, 2010). Our findings were common to both normal and cancer primary sample with some subtle differences. Marginally higher levels of necrosis were observed in the cancer cells following 600-s exposure, yet both samples show almost identical recovery from this treatment (20% surviving fraction). Both normal and cancer cells treated with long LTP exposures (180 and 600 s) undergo autophagy: a completely novel finding in LTP studies on human cells. This may be a survival process for cells that do not undergo necrosis. Our observation of higher levels of autophagy in primary normal cells may be attributed to the hypothesis that normal cells have a higher ROS-threshold tolerance than cancer cells (Gorrini *et al*, 2013).

Although this study argues that LTP could become a potential focal therapy for localised PCa, it remains possible that a reduction in metastatic tumour volume could be observed after treatment with LTP, as a result of necrotic cell death and its associated immune response as outlined earlier. Referred to as spontaneous regression, this response has been documented following necrosis-inducing thermal ablation treatments for other cancers (Sanchez-Ortiz *et al*, 2003; Kim *et al*, 2008; Chu and Dupuy, 2014), but the mechanisms responsible are largely unknown. Nevertheless, a proportion of cells survive LTP treatment and are able to proliferate following exposure to LTP, as demonstrated by their residual colony-forming capacity. The reasons for this must be determined and may potentially be overcome by manipulation and optimisation of the plasma parameters (Cheng *et al*, 2014) and/or pretreatment with a sensitising agent (Frame *et al*, 2013).

Finally, the differences in response we have observed between prostate cell lines and primary cells, particularly in terms of the mechanism of cell death, highlights the importance of studying primary cultures in order to gain an insight into patient efficacy. More specifically, the cell death mechanisms that are triggered following administration of LTP should be elucidated in close-to-patient models.

## Conclusions

In summary, we have clearly demonstrated the potential of LTP as a future therapy option for localised prostate cancer. Through the formation of reactive species (H_2_O_2_ and more than likely also others, e.g., OH, O_2_^−^) in cell culture media, we observed high levels of DNA damage in primary cells cultured directly from patient tissues, together with reduction in cell viability and colony-forming ability. These ultimately lead to necrotic cell death in both normal and tumour samples. However, further optimisation of the LTP operational parameters needs to be conducted, in order to kill the proportion of cells that remain viable after treatment. In addition, although we have previously outlined a potential approach (Hirst *et al*, 2014b), the feasibility of physically treating patients who have PCa with LTP has yet to be established. This would require some modification of the LTP device itself to deliver the LTP to the tumour bed, sparing normal tissues, perhaps employing existing apparatus for cryotherapy and/or brachytherapy.

We believe that with appropriate imaging techniques to facilitate accurate tumour targeting and spare normal tissues, the multi-faceted action of LTP will provide advantages over other focal therapies. More specifically, therapies such as PDT relies on SDO production to destroy cells; plasmas are known to be able produce a multitude of RONS that are toxic to cells. Given that LTPs can be propagated from tubes <100 *μ*m in diameter (Kim *et al*, 2011), we believe that LTP therapy could be more targeted than radiotherapy and more controlled than ice-ball formation in cryotherapy. LTP would not require additional equipment such as the warming catheters used in cryosurgery. Moreover, LTP treatment should prove far more cost-effective than existing treatments.
