# Supplementary figures and images for: Low-temperature plasma treatment induces DNA damage leading to necrotic cell death in primary prostate epithelial cells
Source: Br J Cancer. 2015 Apr 2;112(9):1536–45. doi: 10.1038/bjc.2015.113 (PMC4454887; doi:10.1038/bjc.2015.113)

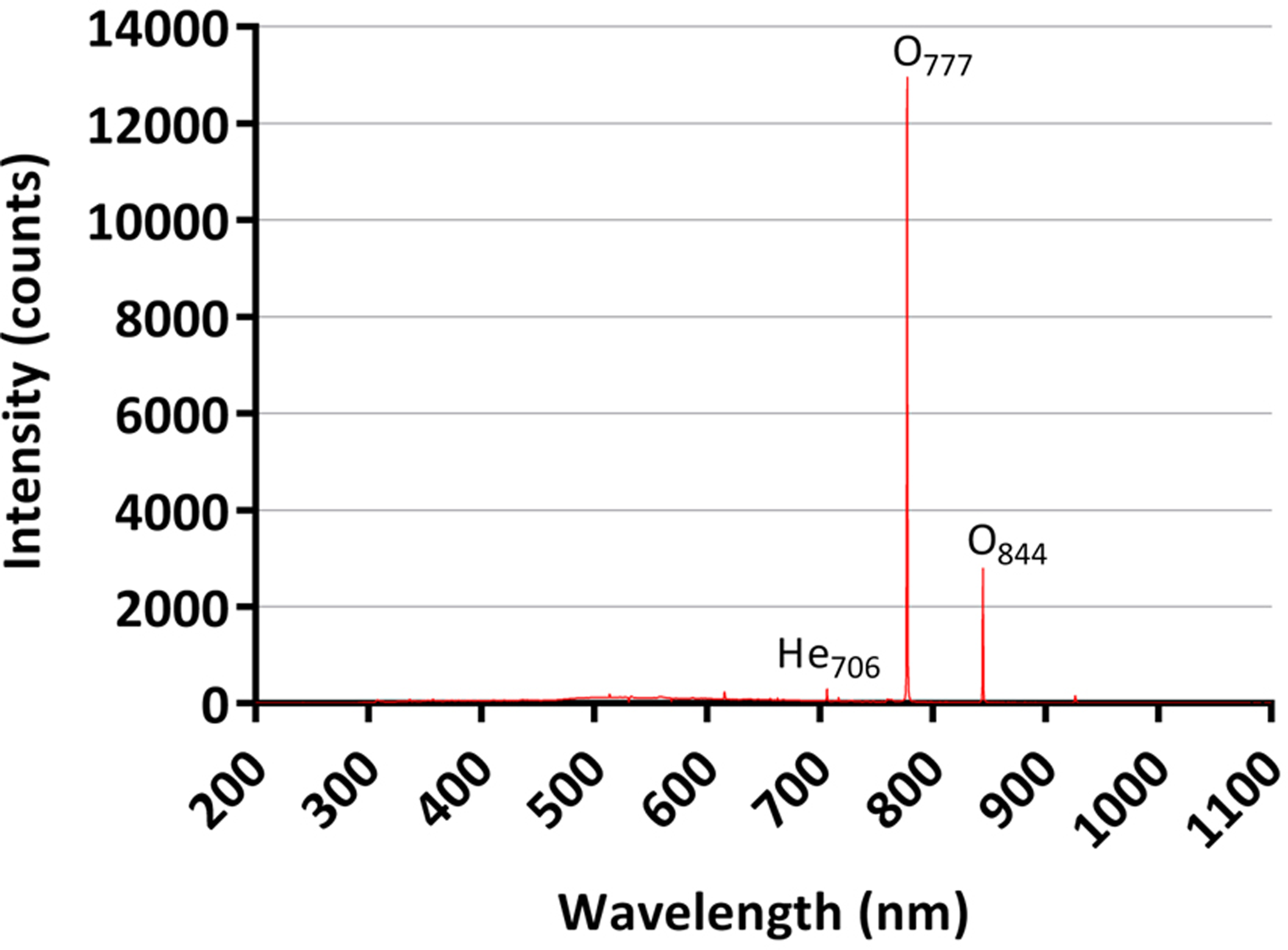

Supplement: Supplementary Figure S1 [file bjc2015113x1.tif]

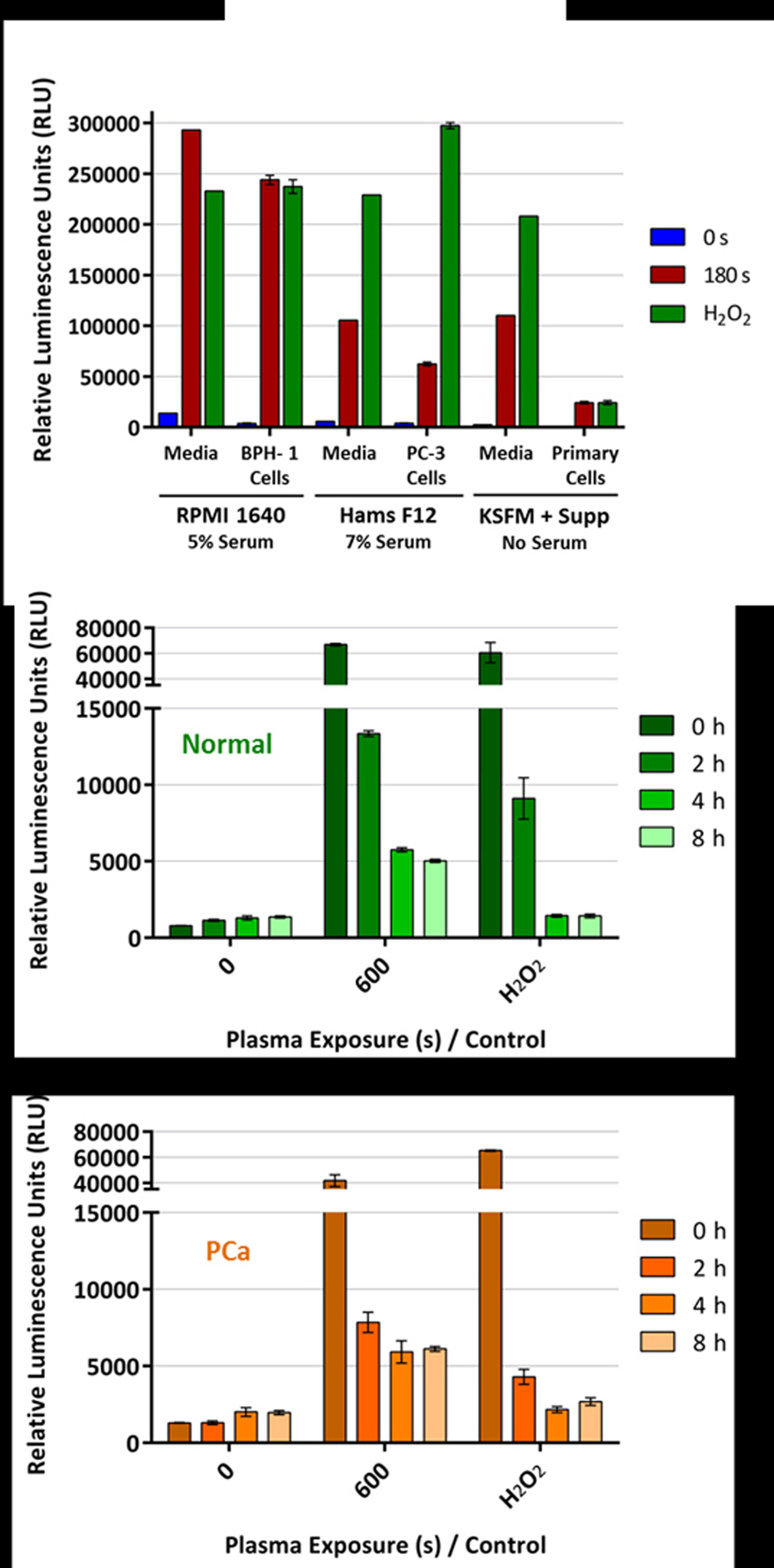

Supplement: Supplementary Figure S2 [file bjc2015113x2.tif]
