# Supplementary material for: Low-temperature plasma treatment induces DNA damage leading to necrotic cell death in primary prostate epithelial cells
Source: Br J Cancer. 2015 Apr 2;112(9):1536–45. doi: 10.1038/bjc.2015.113 (PMC4454887; doi:10.1038/bjc.2015.113)
Supplement: Supplementary Figure Legends [file bjc2015113x3.docx]

**Figure S1. Optical emission spectroscopy of the core plasma.** An optical fibre was aligned directly with the core plasma region and fixed at ~2 cm from the quartz tube. Spectra were obtained using an Ocean Optics HR4000CG-UV-NIR spectrometer (200 – 1100 nm range). Integration time and scans to average were set at 6 s and 50 respectively. A background dark spectrum was obtained and subtracted from subsequent spectra. Peaks characteristic of helium (He, 706 nm) and atomic oxygen (O, 777 nm and 844 nm) are indicated.

**Figure S2. Analysis of hydrogen peroxide formation in cell culture media following plasma treatment.** Immediately following treatment, ROS-Glo H_2_O_2_^™^ luminescence assay (Promega) was performed to ascertain hydrogen peroxide concentration in RPMI 1640 media ± BPH-1 cells, Hams F12 media ± PC-3 cells and keratinocyte *serum-free* media (KSFM, with supplements) ± primary cells (**A**). In addition, the presence of hydrogen peroxide in media at 0, 2, 4, and 8 hours after treatment was also determined for primary normal (**B**) and cancer (**C**) cells. Errors are expressed as mean ± SE.
